# US Farmworkers’ Barriers to Preventing Heat‐Related Illness: An Integrative Review

**DOI:** 10.1002/puh2.222

**Published:** 2024-07-23

**Authors:** Ashley Edgerly, Gordon Lee Gillespie, Beverly M. Hittle, Amit Bhattacharya

**Affiliations:** ^1^ College of Nursing University of Cincinnati Cincinnati Ohio USA; ^2^ Department of Environmental and Public Health Sciences University of Cincinnati Cincinnati Ohio USA

**Keywords:** barriers, farmworkers, occupational health professionals, occupational heat‐related illness, prevention

## Abstract

**Background:**

Occupational heat‐related illness (HRI) is problematic in the United States. Farmworkers are disproportionately affected by HRI compared to other workers. Uncovering barriers that farmworkers face to the prevention of HRI is crucial to reducing HRI‐associated morbidity and mortality. This integrative review aimed to understand US farmworkers’ barriers to preventing HRI.

**Methods:**

An integrative review occurred following Whittemore and Knafl guidelines. Literature searches occurred on PubMed, Medline, and Agricola. After applying inclusion and exclusion criteria and removing duplicates, nine articles remained for review.

**Results:**

There were nine articles in the review. The majority of articles used a convenience sample. In all studies, the populations were farmworkers or agricultural workers. The study designs included cross‐sectional, mixed methods, qualitative focus groups, and a longitudinal study. The articles revealed several themes related to prevention barriers: access to prevention, education and training, work culture, and compensation. Farmworkers often lack access to proper prevention measures, education, and training. Work culture and compensation were obstacles to preventing HRI as some workers feel powerless to speak up for themselves, whereas others are tempted to forgo breaks because of the way they are compensated.

**Conclusions:**

This review indicates the need for more research to understand the barriers farmworkers face to HRI prevention. Providing prevention measures without considering obstacles to their use is ineffective in reducing HRI. Because many farmworkers lack oversight and regulation of prevention measures, focusing on barriers and areas over which farmworkers have more control could have a significant impact.

## Graphical abstract

The results from an integrative review of the literature showed that US farmworkers face barriers to using prevention measures for heat‐related illness. They have insufficient access to prevention and lack education and training. Moreover, work culture inhibits the use of prevention, and the ways that farmworkers are compensated contribute to avoiding prevention measures. Having prevention measures in place is important, but taking an additional step to understand and address any barriers is essential to ensure their effectiveness.

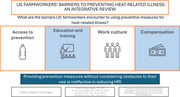

## 1 Introduction

Climate change is expected to raise global temperatures, leading to longer and more intense heat waves [1]. Severe storms and droughts are becoming more frequent, along with increased air pollution and a higher risk of infection [1]. Additionally, it is causing an increase in food insecurity and displacement [1]. Occupational heat‐related illness (HRI) threatens outdoor workers’ health and safety. Exposure to high temperatures and the body's inability to cool down could lead to HRI [2]. Despite ongoing campaigns to reduce illness and mortality, occupational HRI remains a problem [3]. This review sought to fill a gap in the literature to understand farmworkers’ barriers to preventing HRI. The findings from this review could help occupational health professionals guide education, prevention, interventions, and future research to improve worker health.

HRIs are a group of illnesses caused by exposure to heat [4]. HRI may be mild, such as rashes, or more severe, such as heat exhaustion. When not treated, HRI could become life‐threatening. Heat stroke, which can be classic or exertional, is the most dangerous form of HRI [5]. Classic heat stroke typically affects older adults, young children, and those with chronic illnesses and results from exposure to environmental heat beyond what a person's body can regulate. Exertional heatstroke typically affects young and healthy adults. It is the result of a rise in core body temperature from physical activity, sometimes combined with environmental temperatures, that surpasses the body's cooling abilities, resulting in hyperthermia (core temperature above 40°C or 104°F) triggering an inflammatory response with possible multisystem organ failure and death [5, 6].

The individuals most susceptible to HRI include agricultural workers, athletes, firefighters, military personnel, and factory workers [7]. In 2019, there were 3090 reported cases of nonfatal occupational HRIs and 43 deaths related to heat exposure in the workplace [3]. However, it is important to note that these numbers are likely an underestimation due to underreporting [3]. It is also significant to mention that most heat‐related deaths occur during the period from May to September, making the impact on morbidity and mortality even more significant [3].

### 1.1 Prevention Recommendations

The Occupational Safety and Health Administration (OSHA) recommends acclimating workers to heat by following the 20% rule [8]. Other OSHA recommendations include drinking one cup of water every 20 min, taking rest breaks in cool, shaded areas, wearing appropriate PPE (e.g., a hat; light‐colored, loose‐fitting, breathable clothing), and monitoring self and peers for symptoms of HRI [8].

The National Institute for Occupational Safety and Health (NIOSH) also recommends that employers gradually introduce workers to working in hot conditions by following the 20% rule. This involves new workers building up to 100% over 7–14 days and experienced workers starting at 50% and building to 100% over 4 days [9]. NIOSH recommends that employers provide easily accessible and portable clean water, encourage workers to drink one cup of water every 15–20 min, and suggest beverages containing electrolytes when there is heavy sweating [9]. Additional recommendations for employers include encouraging workers to take rest breaks and allowing for modified rest periods, limiting workers’ time in the heat, reducing metabolic demand for employees (e.g., increasing the number of workers for a task, allowing for breaks and a slower pace for high‐exertion tasks, and using tools that can reduce the metabolic load of the employee), training employees on HRI, requiring self‐monitoring, and encouraging the use of the buddy system to monitor coworkers, as well as using heat alert programs [9]. Despite government recommendations, there are no federal heat standards, and only three states have heat standards: California, Minnesota, and Washington [2, 10, 11].

### 1.2 Significance of Prevention of Heat‐Related Illness and Farmworkers

Farm work is distinct in that it is predominantly conducted outdoors, exposing workers to the unpredictable and uncontrollable nature of the climate. This means that individuals working on farms must be prepared to face various weather conditions, including rain, wind, extreme temperatures, and other environmental factors that can impact their work. Additionally, working on a farm often involves strenuous physical labor. Farm work plays a crucial role in ensuring a steady food supply for the nation. Activities such as sowing seeds, tending to crops, and harvesting require timely attention, as each plant has its own unique growth cycle, and unharvested produce can quickly be ruined. This means that the work cannot be postponed, even during periods of extreme heat.

Farmworkers have some of the highest rates of occupational HRI. US crop workers have a 35% higher chance of death than the general workforce as a result of heat stress [8]. However, a lack of HRI recognition could lead to underreporting, indicating the likelihood of much higher numbers [12]. Despite higher rates of HRI and a corresponding increased risk of death among farmworkers, most HRI researchers have focused on military members and athletes [13].

There are gaps and inconsistencies within the current body of literature on farmworkers and their use of HRI prevention measures. For example, there is a lack of understanding as to why farmworkers do or do not perform HRI prevention measures. In one study, researchers recommended that two farmworkers who were found to have high blood pressure seek permission to rest; however, they both refused, and the rationale for the refusal was not discovered [14]. Several studies have found that despite farmworkers receiving training and having knowledge of HRI and proper preventative measures, these measures are still not consistently implemented [15, 16, 17, 18]. Furthermore, it was observed that farmworkers, despite being exposed to high temperatures and the potential risk of HRIs, did not demonstrate concern or awareness regarding the dangers of heat stress [19]. This lack of concern may have implications for their health and safety in the workplace. There could be cultural considerations for specific workplace behaviors. One study revealed that although citrus harvesters frequently experienced symptoms of HRI, they tended to underreport and often did not seek treatment [20]. This reluctance was often attributed to feelings of self‐blame and a prevailing belief that experiencing HRI symptoms was an inherent part of their job [20]. There are differences in prevention behaviors between genders. When comparing male piece‐rate workers to female piece‐rate workers, it was observed that an increase in WetBulb Globe Temperature resulted in a decrease in the work rate of female workers, whereas the work rate of male workers remained unaffected [21].

The higher incidence and increased risk of death from HRI for farmworkers necessitate precedence for research in this area and population. Regardless of prevention measures, the ongoing adverse outcomes from occupational HRI merit exploration. One cause of ongoing HRI could be barriers to the use of the prevention measures put in place to protect farmworkers. This integrative review aimed to identify the barriers US farmworkers encounter to using preventive measures for HRIs.

## 2 Methods

### 2.1 Study Design

The study followed Whittemore and Knafl's five‐step integrative review process: identification, literature search, data evaluation, data analysis, and presentation [22]. Researchers who perform integrative reviews could better understand a phenomenon by including experimental and nonexperimental research.

### 2.2 Search Strategy

**FIGURE 1:**
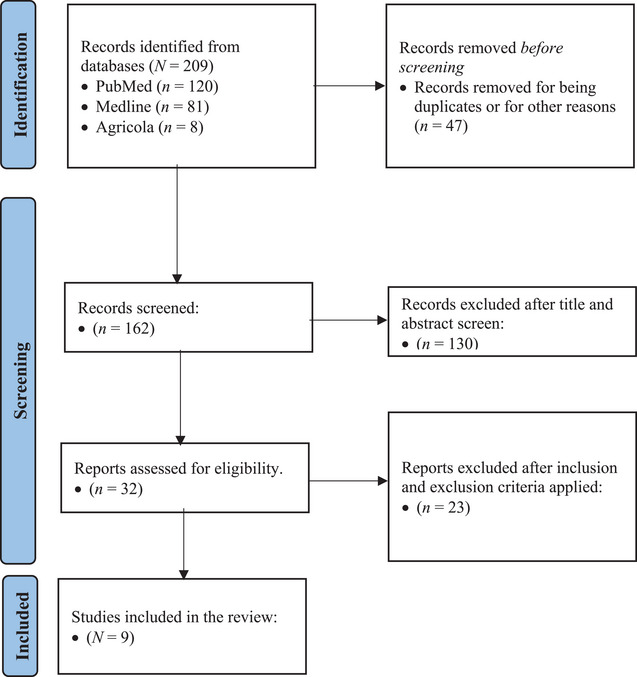
PRISMA Flow Diagram on farmworkers’ barriers to using HRI prevention. *Note*: PRISMA Flow Diagram detailing the screening process for this review.

Inclusion and exclusion criteria were developed before the search. The inclusion criteria were studies performed in the United States, primary research on farmworkers and the prevention of HRI, studies with results that included barriers to prevention, and studies on adults. Articles on farmers or individuals hiring farmworkers were excluded, as their experience may differ from that of hired farmworkers due to power dynamics. (See Table 1 for the full inclusion and exclusion criteria.) This review followed the Preferred Reporting Items for scoping reviews (PRISMA‐ScR) guidelines, and a PRISMA Flow Diagram was used to visually show the screening process for this review (see Figure 1) [23].

**TABLE 1:** Inclusion and exclusion criteria.

Inclusion criteria	Exclusion criteria
Studies conducted in the United States Primary research articles on farmworkers’ prevention of HRI Results that included barriers to prevention Studies on adults 18 years and older English language	Articles on farmers or those hiring farmworkers

*Note*: Table contains the inclusion and exclusion criteria used to assess article eligibility for the review.

Abbreviation: HRI, heat‐related illness.

The health sciences librarian was consulted and assisted with the key terms, and a search for all works occurred in PubMed, Medline, and Agricola prior to February 28, 2021. PubMed was searched using the string ((“Farmers”[Majr] OR farmer*[tiab] OR farmworker*[tiab]) AND (“Accident Prevention”[Mesh] OR “prevention and control” [Subheading] OR “Tertiary Prevention”[Mesh] OR “Primary Prevention”[Mesh] OR prevent*[tiab]) AND (“Heat Stress Disorders”[Mesh] OR heat*[tiab])). Next, there was a search on Medline with Full Text using the string A.B. *heat illness* AND AB (*farmworkers* or *migrant farmworkers* or *agriculture*) AND TX *prevention* AND TX (*barriers* or *obstacles* or *challenges*). Finally, Agricola was searched using the terms *farmworker* AND *heat‐related illness*. There were no limits applied to any of the database searches.

### 2.3 Data Analysis

Relevant information from the nine articles was compiled into a table for data extraction (see Table 2). Subsequent coding of the studies’ findings occurred using Braun and Clarke's six phases for thematic analysis: familiarizing yourself with the data, generating initial codes, searching for themes, reviewing themes, defining and naming themes, and producing the report [24]. Four themes emerged to understand the barriers farmworkers face to using prevention measures: access to prevention, education and training, work culture, and compensation.

**TABLE 2:** Data extraction table.

Citation	Purpose	Methods	Sample, sample size, and setting	Relative study findings	Limitations
Bethel et al. [26]	Compare the hydration and cooling practices between a state with (Washington) and a state without (Oregon) outdoor heat rules	Nonexperimental Cross‐sectional design Surveys developed using existing validated surveys	Convenience sample 100 farmworkers in Oregon 97 farmworkers in Washington During July and August 2013	A greater percentage of participants in Oregon reported that no cooling measures were available at work compared with participants in Washington (40% vs. 5%) Oregon workers more frequently reported the presence of shade structures (29% vs. 5%) and rest stations (19% vs. 8%) Washington more often reported access to shade from trees (92% vs. 47%). 44% of participants reported receiving HRI‐related training: Oregon (54%) and Washington (34%)	No report of reliability or validity of the survey The results may not be generalizable to all crop workers Authors report possible information bias, a small sample size, and a nonrandom sampling method as limitations
Chicas et al. [27]	To understand workers’ perceptions of cooling devices used in the field (cooling vest vs. bandana)	The qualitative portion of mixed methods study Exit interviews used for qualitative data collection	Agricultural workers who included 35 fernery workers 34 nursery workers 10 field crop workers 5 landscape workers 61 participants provided exit interviewsFlorida April–May 2018–2019	Five major themes; two were relevant to HRI prevention barriers: Occupational heat protection practices Majority of workers reporting no protections Some workers reported their employer occasionally provides cool drinking water and ice No worker reported an official employer guideline for drinking water or taking breaks Barriers to implementing occupational heat protection Piece‐rate compensation A lack of interest from employers A lack of regulations to protect them The lack of goodwill by employers to implement OSHA recommendations	Only compared two specific interventions Only 10 workers were farmworkers, and it was not specified how many farmworkers took part in the exit interviews Authors did not list limitations
Fleischer et al. [15]	Determine risk factors that could reduce the risk factors of HIR symptoms among migrant farmworkers in GA	Nonexperimental Cross‐sectional Survey developed based on previous surveys	Convenience sample 405 migrant farmworkers Decatur and Echols counties in South Georgia June 11–23, 2011	No access to regular breaks (34%) No access to shade (27%) Felt they could not negotiate work conditions (work hours and duties)	No mention of reliability or validity Authors’ reported limitations included results represent associations only, limited generalizability, and possible selection bias
Kearney et al. [28]	To better understand the prevalence of HRI and sun safety behavior	Nonexperimental Cross‐sectional design Survey developed using the literature	Convenience sample 158 Latino farmworkers North Carolina August–September 2013	Not all had access to shade, and the majority of access to shade was limited to trees (82.0%), shade structures (21.8%), and rest stations (15.5%)	No reliability or validity of survey Authors reported limitations including small sample size, limited generalizability, and possible response bias
Lam et al. [16]	To identify potential barriers to HRI prevention and treatment in Latino farmworkers	3 Qualitative semistructured focus group discussions and participatory rural appraisal methods Semistructured interviews	Purposive sample 35 Latino farmworkers Washington State Spring 2012	Four themes of potential HRI barriers including: Belief that cooling treatments should be avoided after heat exposure Water location and condition of water affected hydration Caffeinated drinks are preferred to increase work efficiency Belief that sweating causes weight loss	Author‐reported limitations included limited generalizability, possible response bias, and a lack of observations to validate responses
Luque et al. [29]	Describe farmworkers’ experience with HRI prevention strategies and assess HRI information‐seeking preferences, especially the feasibility of using mobile phone apps to access this information	A qualitative pilot study with five structured focus groups	Convenience sample Five focus groups of 6–10 participants 29 Hispanic farmworkers South Carolina October and December 2017	Many only received training on pesticide safety Some work in isolated work areas with no cell phone coverage Misconceptions about the interpretation of not sweating Poor quality of water provided Misconceptions that drinking cold water could make you sick Cooler stocked with beer when they were asked to work longer hours Working with piece‐rate‐based pay limited and shortened their breaks and work too hard When being paid hourly, only the boss decided when farmworkers could take breaks	No mention of reliability or validity Authors did not list any limitations
Luque et al. [19]	To train crew leaders to use the OSHA heat safety tool app and evaluate the utility of the app from a crew leader perspective and to characterize heat safety knowledge, preventive practices, and perceptions of HRI risk among Hispanic farmworkers	Cross‐sectional design Survey adapted from previous studies	Convenience sample 101 Hispanic farmworkers Valdosta, Georgia Between August and mid‐October 2018	88% said they were “very comfortable” taking a break to drink water 19% of participants reported that there was no toilet nearby as one of the reasons for drinking less water 53% were “not at all concerned” about HRI 32% had received some form of HRI training	No mention of reliability/validity Author‐reported limitations included limited generalizability and possible response bias
Mizelle et al. [30]	To explore how sociocultural and occupational factors and environmental heat stress influenced fluid intake and hydration status among Latino farmworkers working in Eastern North Carolina	A community‐informed, sequential exploratory mixed methods studyQualitative data Four focus group discussions with farmworkers Semistructured question guide Quantitative data 32‐item Beverage Intake Assessment Questionnaire (BIAQ) (Validated) Wet bulb globe temperature Urine samples	28 Latino farmworkers (qual) 30 Latino farmworkers (quant) North Carolina. July–August 2020	Two themes were identified: Absence of protection *Subthemes* Intense climate Considerations of workplace exploitation Freedom to drink *Subthemes* Distance and distaste Culture of farmwork	Author‐reported limitations included convenience sampling, small sample size, and potential sampling bias
Stoecklin‐Marois et al. [25]	The purpose of the study was to describe Latino farmworkers understanding about heat‐related illness and how they view their own vulnerability to summer heat conditions	Nonexperimental Part of a larger study; only quantitative follow‐up interviews presented Survey was developed using existing survey instruments and was revised after review and pilot testing in the community	Random sampling 474 Latino farmworkers Mendota, California November 2008–February 2010	87.8% reported employees providing beverages at work site 89.7% able to take a 5‐min break if experiencing heat stress symptoms 93.0% had shaded area available to take a break out of the sun	Author reported limitations included participants self‐reported and due to family recruiting methods, unaccompanied males were excluded

*Note*: Extraction table used to collect and present the data for this review.

Abbreviation: HRI, heat‐related illness.

## 3 Results

The PubMed search yielded 120 results, Medline with Full Text yielded 81 results, and Agricola yielded 9 results. Of the initial 209 articles, 47 were removed as duplicates or unrelated to HRI. A review of the titles and abstracts led to the exclusion of 130 more articles that were not primary research or specific to HRI prevention. A final review of the remaining 32 articles led to the removal of 23 for not having results related to barriers to prevention.

There were nine (*N* = 9) articles in the review. All but two used a convenient sample (*n* = 7), with the remaining using random (*n* = 1) or purposive (*n* = 1) sampling (see Table 2). All but two studies occurred in US states with warmer climates: California (*n* = 1), Florida (*n* = 1), Georgia (*n* = 2), North Carolina (*n* = 2), South Carolina (*n* = 1), Washington (*n* = 1), and a comparison of Oregon and Washington State (*n* = 1). In all studies (*n* = 9), the populations were farmworkers or agricultural workers. Five studies (*n* = 5) were specific to Latino or Hispanic participants, and one (*n* = 1) focused on migrant workers. The study designs varied, with cross‐sectional used the most (*n* = 4), followed by mixed methods (*n* = 2) and qualitative focus groups (*n* = 2). There was one (*n* = 1) longitudinal study design; however, the researchers reported only the exit interview data. All articles’ publication dates were between 2013 and 2022, with four published within the preceding 5 years.

### 3.1 Access to Prevention

Several studies (*n* = 4) found that farmworkers lack access to prevention measures, such as water, shade and breaks, and state and federal regulations [15, 25, 26, 27, 28]. In a study among agricultural workers in 2021, workers reported few employers implementing OSHA's *Water. Rest. Shade*. recommendations due to a lack of state or federal standards [8, 27]. In a study among 158 farmworkers in 2016, it was reported that workers had little access to shade outside of trees [28]. In a study that compared 100 farmworkers in Oregon, a state with no heat standards, to 97 farmworkers in Washington, a state with heat standards, it was found that standards did not always equate to more access [26]. For example, participants in Oregon reported that a lack of cooling measures available at work was more prevalent than in Washington (40% vs. 5%, respectively); however, in Oregon, compared to Washington, workers more frequently reported the presence of shade structures (29% vs. 5%, respectively) and rest stations (19% vs. 8%, respectively), and Washington workers more frequently reported access to shade from trees than Oregon workers (92% vs. 47%, respectively) [26].

When prevention measures are available, access is limited because they are inconvenient or inadequate, such as far away or nonpotable water and drinking supplies [16, 29, 30]. Alternatively, when rules and regulations are in place, they are not always enforced. In a study comparing 100 Oregon farmworkers and 97 Washington farmworkers in 2017, it was found that despite state heat regulations, Washington workers still lacked access to shade [25].

### 3.2 Education and Training

Farmworkers are underprepared to prevent HRI and lack training for what to do if they or a coworker experiences an HRI. The results of several studies suggest that farmworkers have little, inadequate, or no HRI training [19, 26]. Although Washington has state heat rules mandating HRI training for employees, only 34% of participants reported receiving training [26]. In Oregon, where there are no outdoor heat rules, more workers (53%) reported receiving HRI training [26].

This review uncovered several misconceptions, such as it is best to avoid cooling treatments after heat exposure, drinking cool water could cause illness, and sweating or not sweating is not a sign of HRI [16, 29]. During a focus group, one worker reported that sweating or not sweating is not an indication of illness [29]. Another misconception was that some workers associate skin cancer with heat [29].

### 3.3 Work Culture

Work culture emerged as a barrier to prevention in some studies. Some workers felt that they could not speak up or advocate for themselves regarding work conditions, or their breaks were employer‐determined [15, 29]. Another example of work culture as a barrier to HRI prevention emerged in a study among 29 farmworkers in 2019; during focus groups, farmworkers revealed offerings of beer instead of water as an incentive to work overtime [29]. Working in isolation in areas without cell phone coverage was a barrier. In these situations, farmworkers used radios in the case of emergencies [29]. The culture of farmwork was reported as a barrier in a study among farmworkers in North Carolina in 2022 because of associated factors such as poor living conditions without air conditioning or sufficient facilities (e.g., five to six people sharing a bathroom) [30]. Other examples of work culture as a barrier to HRI prevention included that not all workers felt comfortable taking water breaks, and over half of the participants were not concerned about HRI [19].

### 3.4 Compensation

In a qualitative study among agricultural workers in 2021, compensation was identified as a barrier to implementing protection [27]. Piece‐rate workers receive pay based on production, not hours worked. Thus, they are more motivated to skip breaks and overexert themselves to maintain or increase compensation. Similar findings were reported in a qualitative study among 29 farmworkers in South Carolina in 2019, suggesting that piece‐rate pay prevented workers’ use of prevention measures [29].

## 4 Discussion

This review indicates that farmworkers face many barriers to preventing HRI and that farmworkers are often ill‐equipped to prevent HRI due to insufficient education and training. Providing prevention measures and recommendations without considering barriers is an ineffective way to reduce HRI. Prevention measures are essential for farmworkers to reduce their risk of HRI, which may be affected by various factors. Uncovering themes of farmworkers’ barriers to preventing HRI is needed to overcome these barriers. Even with rules and prevention measures, farmworkers continue to get sick and die from the heat. Occupational health professionals facilitating the elimination of barriers to the use of prevention measures would give this population more control over their health and aid in their ability to reduce HRI‐associated risks. This integrative review's outcomes could assist in the future development of education and interventions that consider the unique barriers to HRI prevention for farmworkers. Occupational health professionals are uniquely qualified to protect workers from environmental hazards with a focus on prevention. Because HRI is preventable, occupational health professionals should prioritize research, education, and interventions that focus on prevention.

Inconvenient, inadequate, or a complete lack of access to prevention was a concerning finding in this integrative review. This aligns with the existing knowledge in the field. Even with state regulations in place, basic prevention measures like access to water, rest, and shade, as promoted in OSHA's campaign to prevent HRIs, are often insufficient or inaccessible [8, 15–16, 19, 25–28, 30]. Similarly, workers’ lack of access to prevention is not unique to farmworkers or workers in the United States. Mining workers in Ghana faced HRI prevention barriers due to a lack of specific heat‐related policy regulation [31]. In Australia, access to prevention measures such as shade is only adopted by health safety professionals 35% of the time [32]. Moreover, issues with access to prevention measures extend beyond the workforce. Barriers to implementing heat‐acclimatization guidelines in US high school athletes include limited access to physical resources, such as water and air conditioning [33].

Access to adequate prevention and enforcement of policies that are in place is necessary to avoid HRI. Deaths from HRI continue to rise and were higher in 2019 than in 2012 through 2017 [3]. Although there is some awareness of the dangers that a lack of access to prevention presents, there is more to explore to understand why access remains a problem. In a study of the heat effects on HRI among California farmworkers, researchers found that despite having prevention measures in place, such as water, shade, and breaks, workers did not always use shade provisions or take breaks beyond their lunch break [17]. Thus, there is a need for a deeper understanding of farmworkers’ access to and use of prevention. Policy measures are necessary to ensure access to HRI prevention measures, and a better understanding of the facilitators and barriers to their implementation is vital to ensuring the measures’ use. Procedures should address the factors inhibiting farmworkers from practicing prevention. Occupational health professionals must collaborate with policymakers to establish regulations ensuring the compulsory provision of preventive measures, including access to water, rest, and shade.

A lack of education and training is a barrier to preventing HRI. When workers do not know the signs and symptoms of HRI or which prevention measures to use, they cannot act appropriately to protect themselves. Education prior to starting a job is very important, as 71% of HRI deaths occurred on the day of exposure, suggesting a lack of recognition and timely treatment for HRI [2]. Our findings align with HRI literature, indicating that workers do not always receive HRI training. In one study, workers only received pesticide training [29]. Having the right education can dispel harmful misunderstandings that might otherwise lead to serious misconceptions. Workers may confuse sun safety with HRI safety, such as by associating skin cancer with HRI. Although sun safety and protection from skin cancer are important, HRIs, such as exertional heatstroke, have an immediate risk of death, and workers should be able to differentiate these illnesses. Other misconceptions included beliefs about sweating and cooling [17, 29]. Some workers thought that cooling down too quickly could cause illness; however, rapid cooling is the standard treatment for heatstroke and, when done correctly, has a near 100% survival rate [17, 29, 34]. Another belief is that sweating could lead to weight loss; however, too much sweating without replenishing electrolytes may have deadly outcomes, such as drastically reduced sodium levels (i.e., hyponatremia) [17, 34]. Some participants thought that sweating or not sweating did not indicate HRI. However, one sign of heat exhaustion is heavy sweating, and when exhaustion progresses to heatstroke, sweating may cease, and the person may present with skin that is hot, dry, and red [8, 29]. Workers must receive thorough education and training to equip them with the necessary knowledge and skills to safeguard themselves from HRI and enhance workplace safety. Occupational health professionals can offer training to local healthcare workers responsible for farmworker care, aiming to enhance their ability to identify HRI and administer appropriate treatment.

This integrative review showed that work culture may be a barrier to HRI prevention. Work culture could significantly impact workers’ safety, influencing beliefs, attitudes, and behaviors either positively or negatively [35]. This is consistent with the current literature that, at times, workers are not concerned about HRI [19]. Work culture is a barrier because it affects individuals’ ability to advocate for themselves. For example, some workers feel that they cannot negotiate their working conditions, and not all workers feel comfortable taking water breaks [15, 19]. Although the researchers asked about participants’ comfort, they did not explore the reasons, indicating a need for more research to understand how culture influences safety in HRI behaviors. Promoting a culture of safety and the importance of HRI prevention is needed to reduce the adverse outcomes of harmful work cultures. Occupational health professionals should partner with community members to uncover barriers and work toward creating plans that facilitate farmworkers’ use of HRI prevention. Occupational health professionals could work with community members to develop and disseminate training and education that is accessible and easily understood. Through community collaboration, occupational health professionals could ensure that education and training are culturally appropriate and successfully address work‐culture barriers to prevention. Moreover, health care should extend beyond HRI treatment to prevention. HRI is a recent inclusion in health textbooks; nursing and medical curricula generally focus on treating individual HRI [12]. Occupational health professionals could lead the way in creating a holistic curriculum that focuses on prevention.

This study indicated that compensation could be a barrier to HRI prevention for farmworkers. This is consistent with the literature. For example, there is evidence that some piece‐rate workers overexert themselves and skip or shorten their breaks, as low output affects their wages and negative effects on income cause some workers to avoid breaks [2, 36]. Workers’ ability to rest or slow their work rate is crucial to prevent exertional HRI. Policy changes are needed to protect workers from compensation systems that put them at risk. Occupational health professionals may play an important role in increasing equity and justice for farmworkers by advocating for fair wages, affordable and adequate housing, and other fundamental human rights, such as healthcare access. Moreover, health care should extend beyond HRI treatment to prevention. Increased access to affordable housing may be helpful, as farmworkers who are not reliant on employer‐provided accommodations could take fewer risks and have more employment choices.

## 5 Limitations

There were several limitations to this review. Only one study aimed to discover barriers to HRI prevention, indicating a lack of research specific to the phenomenon. Therefore, the results of this integrative review may not be conclusive. However, to expand the knowledge on barriers to prevention and minimize this limitation, studies that produced findings on barriers were included in the review. This review was conducted by only one reviewer; however, a librarian was consulted and assisted with the search. Another limitation is that the population varied in each study. For example, in the reported data from a study among different types of agricultural workers, the researchers did not specify how many of the 61 exit interview participants were field workers [15]. However, due to the lack of research specific to the farmworker population, including these studies to add to the knowledge about HRI issues was important. Finally, the integrative review's results may not be generalizable to all farmworkers, because the studies included a limited number of states; however, this selection was unavoidable due to the lack of research conducted in other states.

## 6 Conclusion

The results of this study revealed that farmworkers face many barriers to using HRI prevention measures that are intended to keep them safe. These barriers included a lack of access to proper prevention measures, education, and training. Other barriers include work culture and compensation. Workers deserve access to potable water, breaks, and shade. They also deserve to feel safe to advocate for themselves. In the nine reviewed studies, there was limited time spent with the farmworkers, with minimal opportunity for them to voice their opinions or expertise on the issues of HRI, prevention measures, or barriers to prevention. Even with rules and prevention measures in place, farmworkers continue to experience symptoms of and die from HRI. Eliminating barriers to the use of prevention measures could allow this population to have more control over their health and aid in their ability to reduce HRI‐associated risks.

Future research should employ qualitative research methodologies such as ethnography and spend more time engaging with farmworkers to obtain firsthand insights into HRI prevention and the barriers they face. This approach could yield invaluable results and contribute to the development of culturally sensitive educational initiatives and interventions tailored to this population's needs. It is also vital to shift the focus toward areas where farmworkers have more control, especially in instances where oversight and regulation of prevention measures are lacking. Many farmworkers lack oversight and regulation of prevention measures; therefore, focusing on barriers and areas over which farmworkers have more control could have a more significant impact.

## Author Contributions

Ashley Edgerly designed and conducted the review. Gordon Gillespie, Beverly Hittle, and Amit Bhattacharya all contributed to drafting and revising the manuscript.

## Ethics Statement

The authors have nothing to report.

## Conflicts of Interest

The authors declare no conflicts of interest.

## Data Availability

The data that support the findings of this study are available in the supplementary material of this article.
